# Machine Learning Models to Predict Survival Outcomes According to the Surgical Approach of Primary Radical Hysterectomy in Patients with Early Cervical Cancer

**DOI:** 10.3390/cancers13153709

**Published:** 2021-07-23

**Authors:** Se Ik Kim, Sungyoung Lee, Chel Hun Choi, Maria Lee, Dong Hoon Suh, Hee Seung Kim, Kidong Kim, Hyun Hoon Chung, Jae Hong No, Jae-Weon Kim, Noh Hyun Park, Yong-Sang Song, Yong Beom Kim

**Affiliations:** 1 Department of Obstetrics and Gynecology, Seoul National University College of Medicine, Seoul 03080, Korea; seikkim1@snu.ac.kr (S.I.K.); marialee@snu.ac.kr (M.L.); bboddi0311@snu.ac.kr (H.S.K.); chhkmj1@snu.ac.kr (H.H.C.); kjwksh@snu.ac.kr (J.-W.K.); pnhkhr@snu.ac.kr (N.H.P.); yssong@snu.ac.kr (Y.-S.S.); 2 Department of Genomic Medicine, Seoul National University Hospital, Seoul 03080, Korea; sy@snuh.org; 3 Samsung Medical Center, Department of Obstetrics and Gynecology, Sungkyunkwan University School of Medicine, Seoul 06351, Korea; chelhun.choi@samsung.com; 4 Department of Obstetrics and Gynecology, Seoul National University Bundang Hospital, Seongnam 13620, Korea; sdhwcj@snubh.org (D.H.S.); kidong.kim.md@snubh.org (K.K.); jhno@snu.ac.kr (J.H.N.)

**Keywords:** cervical cancer, hysterectomy, minimally invasive surgery, laparoscopy, survival rate, recurrence, machine learning

## Abstract

**Simple Summary:**

An increased risk of relapse and death from minimally invasive radical hysterectomy has been reported in some patients with early cervical cancer. Thus, the development of an intuitive and precise decision-aid tool, which estimates recurrence and mortality rates by surgical approach, is necessary. To develop models predicting survival outcomes according to the surgical approach, we collected clinicopathologic and survival data of patients with 2009 FIGO stage IB cervical cancer who underwent a radical hysterectomy. Using only variables that could be obtained preoperatively, we developed various models predicting the probability of 5-year progression-free survival and overall survival. Among them, hybrid ensemble models, combined with logistic regression and multiple machine learning models, achieved the best predictive performance. The developed models are expected to help physicians’ and patients’ decision making related to the surgical approach for primary radical hysterectomy.

**Abstract:**

We purposed to develop machine learning models predicting survival outcomes according to the surgical approach for radical hysterectomy (RH) in early cervical cancer. In total, 1056 patients with 2009 FIGO stage IB cervical cancer who underwent primary type C RH by either open or laparoscopic surgery were included in this multicenter retrospective study. The whole dataset consisting of patients’ clinicopathologic data was split into training and test sets with a 4:1 ratio. Using the training set, we developed models predicting the probability of 5-year progression-free survival (PFS) and overall survival (OS) with tenfold cross validation. The developed models were validated in the test set. In terms of predictive performance, we measured the area under the receiver operating characteristic curve (AUC) values. The logistic regression models comprised of preoperative variables yielded AUCs of 0.679 and 0.715 for predicting 5-year PFS and OS rates, respectively. Combining both logistic regression and multiple machine learning models, we constructed hybrid ensemble models, and these models showed much improved predictive performance, with 0.741 and 0.759 AUCs for predicting 5-year PFS and OS rates, respectively. We successfully developed models predicting disease recurrence and mortality after primary RH in patients with early cervical cancer. As the predicted value is calculated based on the preoperative factors, such as the surgical approach, these ensemble models would be useful for making decisions when choosing between open or laparoscopic RH.

## 1. Introduction

Cervical cancer is the fourth most common female cancer for both incidence and mortality, with an estimated 604,127 new cases and 341,831 cancer deaths worldwide in 2020 [1]. There are significant geographical variations in cervical cancer incidence and mortality rates. In Korea, despite a gradual decrease in incidence [2], the proportion of annual cervical cancer among newly diagnosed female cancers remains higher, compared to the United States (2.5% vs. 1.6%) [3,4]. The presence of early symptoms and effective screening tools have led to more than a third of cervical cancer cases being diagnosed at an early stage [4]. For early cervical cancer, primary radical hysterectomy (RH) is recommended [5,6].

RH has been commonly performed via minimally invasive surgery (MIS) [7,8]. However, this approach declined in popularity after the publication of “Laparoscopic Approach to Carcinoma of the Cervix (LACC)”, a phase III randomized controlled trial (RCT), in 2018 [9]. This trial reported an increased risk of relapse and death in MIS RH versus conventional open RH (ORH) in patients with the 2009 International Federation of Gynecology and Obstetrics (FIGO) stage IA1 (lymphovascular invasion [LVSI]) to IB1 lesions [9]. Subsequent retrospective studies from different study groups also reported inferior survival outcomes with MIS RH [10,11,12,13,14,15,16,17].

Before abandoning MIS RH in all patients with early cervical cancer, there was an effort to find a specific population that can safely undergo MIS RH without compromising survival outcomes. Previously, our research team suggested patients with 2009 FIGO stage IB1 and cervical mass size ≤ 2 cm on preoperative magnetic resonance imaging (MRI) were safe candidates as laparoscopic RH (LRH) did not influence disease recurrence in this subgroup [13,14]. This subgroup also had a similar recurrence rate, regardless of surgical approach in the SUCCOR study, a European retrospective cohort study [11].

Further well-designed confirmatory prospective studies are warranted to identify optimal candidates for MIS RH. Until then, physicians must discuss with their early cervical cancer patients regarding surgical approach plans for RH [18]. It would thus be a great help if an intuitive and precise decision-aid tool, which estimates recurrence and mortality rates by surgical approach, is developed. Recently, machine learning methods have found popular applications in clinical cancer research, especially for predicting cancer prognosis [19]. However, solo use of machine learning models might not be much more beneficial than traditional logistic regression [20,21]. Therefore, we believe that a combination of both logistic regression and machine learning models is required to achieve higher predictive capabilities.

Thus, this study aimed to develop hybrid ensemble models predicting the risk of disease recurrence and mortality according to the surgical approach in FIGO stage IB cervical cancer patients. Focusing on clinical utility, we only used factors or variables, which could be identified preoperatively.

## 2. Materials and Methods

### 2.1. Study Population

Inclusion criteria for the study population were as follows: (1) patients with 2009 FIGO stage IB cervical cancer; (2) either primary laparoscopic or open Type C RH procedures, as defined in the Querleu and Morrow classification [22] at the three included hospitals between 2000 and 2018; (3) preoperative MRI scans were available. We excluded patients with any of the following conditions: (1) received chemotherapy or radiation preoperatively; (2) histologic types other than squamous cell carcinoma, usual type adenocarcinoma, and adenosquamous carcinoma; (3) insufficient clinicopathologic data.

From the cervical cancer cohorts of the three institutions in Korea, a total of 1138 patients who met these inclusion and exclusion criteria were identified. Among them, we further selected patients by assessing treatment adequacy to develop more accurate predictive models. We excluded 82 patients who did not receive adjuvant radiation although they belonged to either pathologic high-risk group (at least one of the three pathologic findings, positive resection margin, parametrial [PM] invasion, and lymph node (LN) metastasis) or intermediate-risk group (according to the Sedlis criteria [23]).

### 2.2. Data Collection

During the study period, all surgical procedures were performed by faculty who had completed their gynecologic oncology fellowship training. Before the LACC trial reports, no internal policies existed for selecting an optimal surgical approach for primary RH in patients with early cervical cancer. From the medical records and pathologic reports review, we collected information about clinicopathologic characteristics, such as age, stage, preoperative conization, surgical procedures, pathologic findings, and adjuvant treatments.

In addition to preoperative MRI, patients frequently underwent computed tomography (CT) scans and/or positron emission tomography (PET) to evaluate nodal and distant site metastasis. We reviewed imaging studies taken within one month prior to surgery and assessed the following: First, we measured the cervical mass size on preoperative MRI, as MRI has been shown to be the most accurate imaging modality for this [24]. Second, patients suspected of PM invasion were identified using preoperative MRI, as MRI is the best modality to evaluate the local extent of cervical cancer [25,26,27,28]. Third, all the available imaging modalities for assessing pelvic and para-aortic LN status were evaluated.

After primary treatment, surveillance methods were the same among the three institutions. Regardless of surgical approach, routine CT scans were conducted every 3–4 months for the first two years, every half year for the next two years, and then, every year or when the patients were suspected of recurrence by symptoms or abnormal examination findings. We determined disease progression according to the Response Evaluation Criteria in Solid Tumors version 1.1 [29]. For survival data, progression-free survival (PFS) was calculated from the date of RH to the date of disease progression, while overall survival (OS) was calculated from the date of RH to the date of cancer-related death or last follow-up.

### 2.3. Dataset Preprocessing

Statistical analyses were performed using IBM SPSS statistics software (version 25.0; IBM Corp., Armonk, NY, USA) and R statistical software (version 4.0.2; R Foundation for Statistical Computing, Vienna, Austria). Unless otherwise stated, a *p* value less than 0.05 was considered statistically significant.

**Figure 1:**
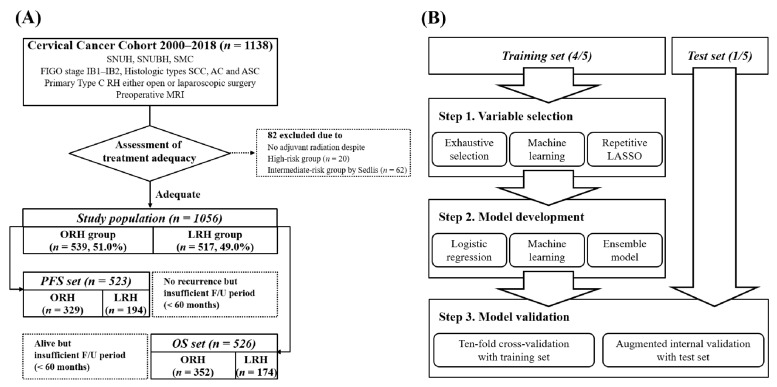
Flow diagrams of this study: (**A**) selection of the study population; (**B**) consecutive processes of model construction.

We set the outcomes of the predictive models as the probabilities of 5-year PFS and OS. Thus, we filtered the study population by the following: patients without recurrence and with <60 months follow-up were excluded from PFS set, while those who were alive with <60 months follow-up were excluded from the OS set (Figure 1A).

As shown in Figure 1B, the whole process consisted of three steps: variable selection, model development, and model evaluation. PFS and OS sets were separated into training and test sets with a 4:1 ratio. Variable selection and model development were conducted in the training set, and the developed models were validated in the test set. We imputed all missing data using a multiple imputation approach, using R package mice (version 3.13.0).

### 2.4. Variable Selection

The following 13 preoperative variables were initially included: institution, age, surgical approach, 2009 FIGO stage, histologic type, preoperative conization, serum levels of three tumor markers (CEA, SCC, and CA-125), cervical mass size measured by MRI, suspicious for PM invasion evaluated by MRI, and LN status evaluated by pretreatment imaging studies, and LVSI. To minimize selection bias, we did not apply any constraints during the variable selection step. From the distributions of serum CEA, SCC, and CA-125 levels, their log-transformed values were also considered as candidate predictors. In addition, tumor size was binarized using 20 mm as the cutoff value to compensate predictor’s quantitative effect. We set 0.1 as the significance level of the statistical analyses to incorporate more putative predictors into the model development process.

We evaluated three scenarios of variable selection to minimize overfitting and multicollinearity: (1) exhaustive variable selection; (2) machine-learning-based variable selection; (3) LASSO-based resampling methods. In exhaustive variable selection, all possible combinations of four to eight variables were investigated to identify the model whose predictors had individual statistical significance. For the machine-learning-based approach, we measured the importance value for each available predictor that quantified the method-specific contribution of the predictor to the prediction model using R package caret (version 6.0-86). The variable selection was achieved by summing the importance values from four different machine learning methods (random forest, decision tree, neural network, and support vector machine) and prioritizing them. Lastly, LASSO-based variable selection was performed by selecting the variables whose selection probability was more than 50% in the resampled replicates of the original dataset.

### 2.5. Model Development and Validation

We constructed multiple predictive models using the selected predictors from each strategy. We considered three different methods for building the predictive models: (1) logistic regression methods; (2) machine learning methods with a multitude of different approaches; (3) ensemble methods that integrated the aforementioned two methods. In this step, a total of eight machine learning approaches were used (Table S1). Prediction performance evaluation was carried out using the area under the receiver-operating characteristic curve (AUC) values, and statistical significance was evaluated using DeLong’s test that compared two AUCs from a dataset under the null hypothesis that two AUCs were the same. 

To minimize bias, the developed predictive models were validated using two schemes: tenfold cross validation (CV) and augmented internal validation (IV). The augmented IV performed repeated evaluation using an identical scheme in the model development step. Trained models were applied to the test set to measure the predictive performance. To further reduce putative bias, such as overfitting or confounders, both CV and IV were stratified by an institution and surgical approach.

For the development of the hybrid ensemble model, machine learning methods, which were individually trained using the training set, were combined with the logistic regression model to identify an optimal combination of the predictive models. Up to four models were combined with weights *w* (*w* = 0, 0.1, …, 0.9, 1, Σ*w* = 1). We tested all four-way combinations for the nine models (logistic regression and eight machine learning models) with all possible combinations of weights.

## 3. Results

### 3.1. Patient Characteristics

**Figure 2:**
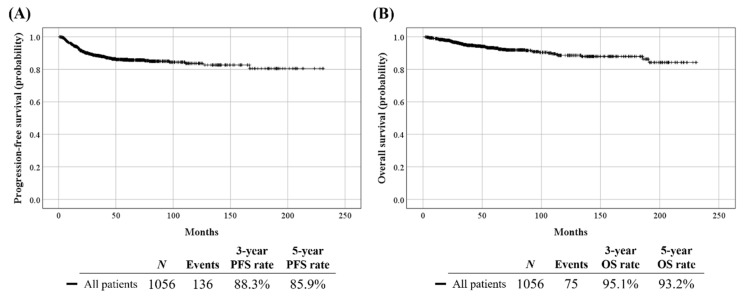
Survival outcomes of the study population: (**A**) PFS; (**B**) OS.

Table S2 depicts patient clinicopathologic characteristics (*n* = 1056). Among the study population, 369, 168, and 519 were included from Seoul National University Hospital, Seoul National University Bundang Hospital, and Samsung Medical Center, respectively. Preoperative conization was administered in 32.9% of patients. The median cervical mass size on preoperative MRI was 22.0 mm. By preoperative imaging studies, 24.4% and 14.6% were suspected of PM invasion and LN metastasis, respectively. The median length of observation was 57.9 months during which 136 (12.9%) experienced disease recurrence and 75 (7.1%) died (Figure 2).

**Figure 3:**
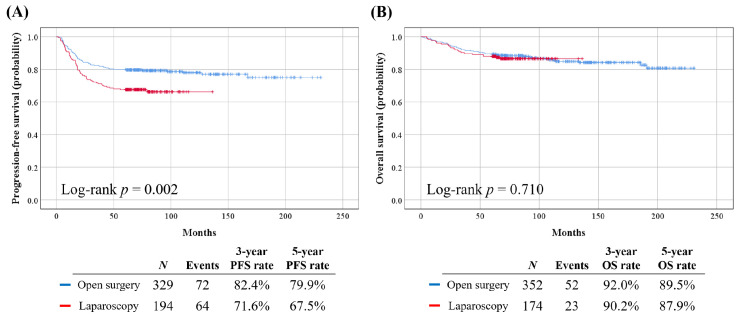
Comparisons of survival outcomes between the LRH and ORH groups in the PFS and OS sets: (**A**) PFS; (**B**) OS.

Patient clinicopathologic characteristics in the PFS set (*n* = 523) and OS set (*n* = 526) are presented in Table 1. Among patients in the PFS set, those who underwent LRH showed significantly worse PFS, compared to those who underwent ORH (5-year PFS rate, 67.5% vs. 79.9%; *p* = 0.002). However, in the OS set, no significant difference was observed regarding OS between the LRH and ORH groups (*p* = 0.710) (Figure 3).

### 3.2. Variable Selection

The exhaustive variable selection methods yielded the following six variables for predicting five-year PFS rate: surgical approach, serum levels of CEA and SCC, preoperative conization, 2009 FIGO stage, and LN status on imaging studies. For predicting the five-year OS rate, the following six variables were identified: serum levels of CEA, SCC, and CA-125, LN status on imaging studies, cervical mass size by MRI, and histologic type. The log-transformed variables were not selected (Table 2). The machine learning methods tended to select continuous variables (e.g., log(SCC) and cervical mass size by MRI) or qualitative variables (e.g., histologic type), instead of binary or the binarized variables (Figure S1). The LASSO-based resampling method with 1000 resampled replicates selected eight variables (age, surgical approach, log(SCC), LVSI, preoperative conization, 2009 FIGO stage, cervical mass size by MRI, and histologic type) for predicting both 5-year PFS and OS rates, but the individual significance for each predictor was insufficient. Consequently, we decided to use the variables selected by the exhaustive variable selection method in the subsequent analyses.

### 3.3. Model Development and Validation

**Figure 4:**
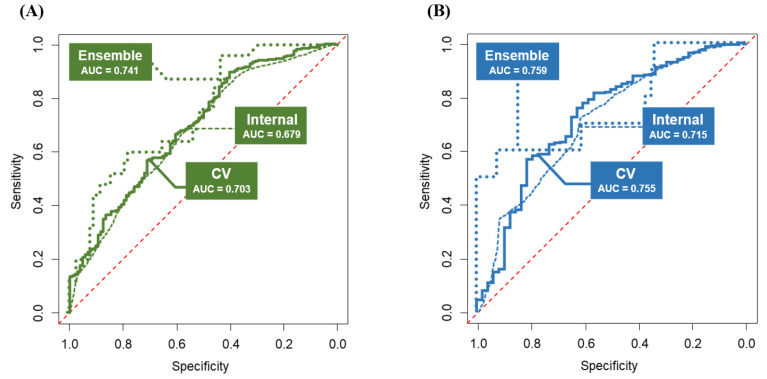
Predictive performance of the developed models. The logistic regression models underwent tenfold cross validation (presented as line) and internal validation with 20% of predivided test set (presented as dash). The ensemble models also underwent internal validation with 20% of predivided test set (presented as dots): (**A**) ROC curves with the AUC values for 5-year PFS; (**B**) ROC curves with the AUC values for 5-year OS.

**Figure 5:**
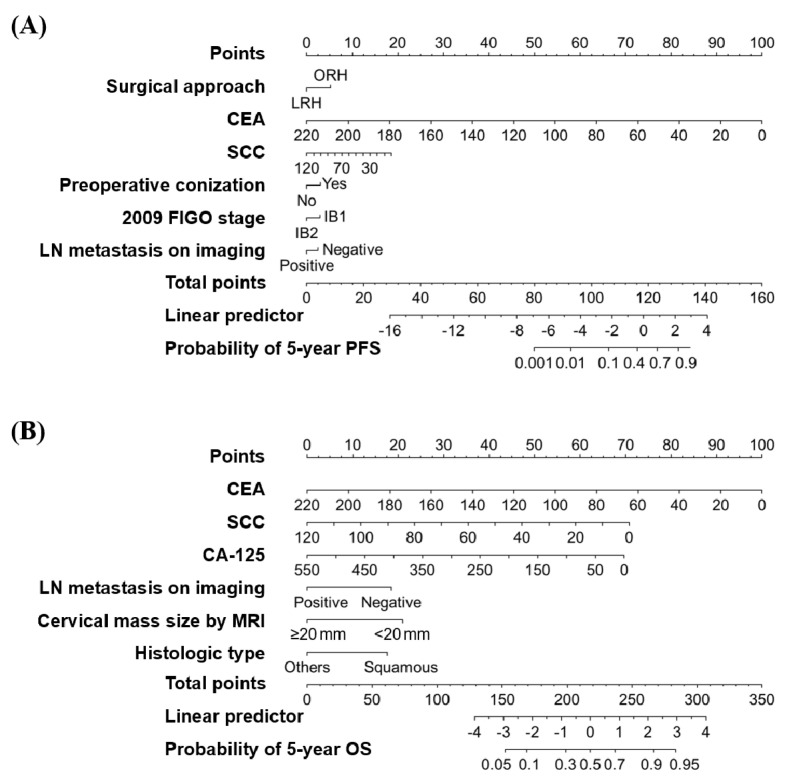
Logistic-regression-based nomograms predicting probabilities of (**A**) 5-year PFS and (**B**) 5-year OS.

The logistic regression models showed 0.703 and 0.755 for AUCs in tenfold CV for predicting probabilities of 5-year PFS and OS, respectively (Figure 4). We also performed augmented IV using the test set. As regards the results, the predictive performance of the logistic regression models decreased slightly (AUCs, 0.679 and 0.715 for 5-year PFS and OS, respectively) but showed no significant differences regarding AUCs (DeLong’s test *p* > 0.05), suggesting stability of the predictive models. The developed logistic-regression-based nomograms for the prediction of 5-year PFS and OS rates are shown in Figure 5.

The machine learning models showed questionable predictive performance. Even in the best scenario, prediction powers were bounded to 0.58 and 0.61 in AUCs for predicting 5-year PFS and OS rates, respectively, with statistically significant differences (DeLong’s test *p* < 0.01).

The hybrid ensemble models were developed by putting logistic regression and machine learning models together. The ensemble models showed much improved predictive performance (AUCs, 0.741 and 0.759 for predicting probabilities of 5-year PFS and OS, respectively) (Figure 4). For the 5-year PFS rate, the best model consisted of naïve Bayes and gradient boosting machine learning models with weights of 0.8 and 0.2, respectively. For the 5-year OS rate, the best model consisted of logistic regression, naïve Bayes, and neural network models with weights of 0.6, 0.3, and 0.1, respectively. We also measured the effect of imputation to assess whether the imputation process affected the prediction powers. For 5-year PFS and OS rates, the ensemble models were evaluated in 110 and 101 complete patients, respectively. Consequently, they showed 0.776 and 0.767 regarding AUCs for 5-year PFS and OS, respectively.

Lastly, we set up a website (http://lsy.io/nomogramECC, accessed on 1 June 2021) implementing logistic-regression-based nomograms and hybrid ensemble models to facilitate clinical use. Users can input nine risk factors using the web interface and see the processes of calculating with 5-year PFS and 5-year OS nomograms. The calculated prediction values from the logistic-regression-based and hybrid ensemble models are presented in parallel.

## 4. Discussion

Herein, more than a thousand patients with FIGO stage IB cervical cancer who received RH by either laparoscopic approach or conventional open surgery at three tertiary institutional hospitals were reviewed. Using multidimensional variables, encompassing both clinicopathologic factors and serum tumor markers and imaging studies, we successfully developed preoperative models predicting survival outcomes according to the surgical approach. The hybrid ensemble models combining logistic regression and multiple machine learning models showed better performance in predicting 5-year PFS and OS rates than the logistic regression models.

In line with previous observational studies after the LACC trial [10,11,12,13,14,15,16,17], we also demonstrated that LRH was associated with worse PFS, compared to ORH. Selected as a prognostic factor for disease recurrence, the surgical approach for RH was incorporated in the developed models and nomograms predicting the probability of 5-year PFS. Interestingly, preoperative conization was selected as a favorable prognostic factor for PFS. As we discussed previously, conization might reduce the primary cervical mass size and subsequent potential for tumor spillage [13].

In contrast, LRH and ORH showed similar OS in this study. Consistently, the surgical approach was not selected as a prognostic factor for OS by exhaustive variable selection methods. We recognize that the LACC trial [9], retrospective cohort studies from Melamed et al. [10] and other research groups [11,12], and a recent meta-analysis study [30] commonly reported that MIS RH was associated with worse OS in patients with early cervical cancer. However, similar to this study, retrospective cohort studies from our research group [13,14] and others [15,16,17] reported that MIS RH did not increase the risk of death, compared with ORH.

Since counseling with regard to the surgical approach is usually conducted before surgery, only preoperative variables were used in this study. However, the number of available preoperative variables was initially limited; thus, we also collected data from imaging studies (cervical mass size, PM invasion, and LN status). Similar to our previous study results [13,14], the study population herein was divided based on MRI-determined cervical mass 2 cm, which was also reflected in our developed models and nomograms. For assessing LN metastasis, MRI, CT, and PET/CT perform similarly, with high specificity but low sensitivity [28]. Nevertheless, LN status on preoperative imaging studies was incorporated in our developed models predicting probabilities of 5-year PFS and 5-year OS. Nowadays, imaging studies have become essential for the pretreatment assessment of cervical cancer, as embodied in the 2018 FIGO staging system [31].

Among the study population, PM invasion and LN metastasis were pathologically confirmed in 14.4% and 20.9%, respectively. According to the results of ABRAX, an international retrospective cohort study, the completion of RH after intraoperative confirmation of LN metastasis did not improve PFS and OS in patients with 2009 FIGO stage IA-IIB cervical cancer [32]. Therefore, for early cervical cancer patients suspected of LN metastasis on preoperative imaging studies, checking intraoperative LN assessment first, and then, according to the results, considering abandoning the RH procedure might be one of the reasonable therapeutic options. However, such a strategy was not considered in the current study; instead, all patients underwent RH universally.

In the era of precision cancer medicine, precise prediction of prognosis after a certain treatment is a very important issue. Learning from a large amount and variety of data, machine learning techniques enable detecting hard-to-discern patterns from large, noisy, or complex data sets [33]. As the quality of the data is important in the development of machine learning models, we excluded patients who were out of the guidelines and treated improperly. Nevertheless, the predictive performance of the machine learning models was inferior, compared to those of logistic regression models. Such inferiority might originate from an insufficient sample size for developing predictive models and a considerable number of missing values for some variables, such as preoperative serum tumor markers. To solve the latter, we had no choice but to impute all the missing data.

In this study, the hybrid ensemble models combining logistic regression and multiple machine learning models showed better predictive performance than the machine learning models and logistic regression models alone. This suggests two considerations: First, the ensemble models achieved predictive superiority by averaging the predictive performance of both logistic and machine learning models and by reflecting both linear and nonlinear relationships across multiple identified predictors. Second, more preoperative variables should be discovered and incorporated into existing models for better prediction of survival outcomes. For example, expressions of Ki-67 and other proteins, and microRNA expression signatures of tissue, obtained from biopsy or conization, might serve as powerful prognostic biomarkers in patients with early cervical cancer [34]. If these molecular biomarkers are combined with macroscale clinicopathologic data, the robustness and accuracy of prognostication might improve considerably.

For the clinical use of our developed models, we implemented a web-based system. We recognize that there are some differences between variables, predicting 5-year PFS and OS rates. For example, the surgical approach for RH was incorporated in the developed models predicting the probability of 5-year PFS but not for 5-year OS. However, PFS and OS cannot be considered separately during patient counseling. In this aspect, users are asked to input nine risk factors: surgical approach, serum levels of CEA, SCC, and CA-125, preoperative conization, 2009 FIGO stage, LN status on imaging studies, cervical mass size by MRI, and histologic type. And then they obtain the four prediction values simultaneously: the probabilities of 5-year PFS and OS calculated by logistic-regression-based and hybrid ensemble models. As it shows these results on one screen, this system would be an individualized, useful source for consultation between physicians and patients. Users can also estimate how much specific variable or factor in the logistic regression models affects the 5-year PFS and OS rates: in particular, our web-based nomograms visualize this intuitively. Thus, we believe that it will help physicians’ and patients’ decision making when choosing between LRH and ORH. In contrast, although hybrid ensemble models showed better predictive performance than the logistic-regression-based models, it is difficult to know precisely each variable’s contribution to the prognosis owing to the machine learning components.

The current study had several limitations. First, inter-institutional heterogeneity might exist (e.g., differences in patients’ characteristics and detailed procedures of surgery). Second, we did not investigate intracorporeal colpotomy and the use of uterine manipulators, both known to be associated with risk factors for disease recurrence in MIS RH [35]. Third, surgery-related complications or quality of life issues, fertility-sparing surgery, and less radical surgery concepts were not considered in this study. Lastly, robot-assisted RH cases were excluded, as robotic RH remains uncommon due to its high costs in Korea.

## 5. Conclusions 

In conclusion, we successfully developed logistic-regression-based and hybrid ensemble models predicting disease recurrence and mortality after primary RH in patients with 2009 FIGO stage IB cervical cancer, according to the surgical approach. As the developed predictive models consisted of only variables that could be obtained preoperatively and implemented in a website with a user-friendly interface, they would be helpful to patients and physicians for making decisions related to the surgical approach for primary RH. Further external validation studies with larger samples are warranted.

## Figures and Tables

**Table 1:** Clinicopathologic characteristics of PFS and OS sets.

Characteristics	PFS Set (*n* = 523, %)	OS Set (*n* = 526, %)
Age, years	47.6 (24.6–78.1)	48.0 (24.6–78.1)
Surgical approach		
Open surgery	329 (62.9)	352 (66.9)
Laparoscopy	194 (37.1)	174 (33.1)
2009 FIGO stage		
IB1	442 (84.5)	452 (85.9)
IB2	81 (15.5)	74 (14.1)
Histologic type		
Squamous cell carcinoma	377 (72.1)	393 (74.7)
Adenocarcinoma	116 (22.2)	105 (20.0)
Adenosquamous carcinoma	30 (5.7)	28 (5.3)
Preoperative conization		
No	364 (69.6)	355 (67.5)
Yes	159 (30.4)	171 (32.5)
Preoperative tumor markers		
CEA, ng/mL	1.3 (0.1–210.0) ^a^	1.3 (0.1–210.0) ^d^
SCC, ng/mL	1.0 (0.1–118.7) ^b^	1.0 (0.1–118.7) ^e^
CA-125, IU/mL	12.1 (0.9–271.5) ^c^	12.0 (0.9–273.0) ^f^
Cervical mass size by MRI, mm	22.0 (0–82.0)	20.5 (0–82.0)
No residual tumor	134 (25.6)	148 (28.1)
<20	83 (15.9)	86 (16.3)
≥20 and <40	200 (38.2)	195 (37.1)
≥40	106 (20.3)	97 (18.4)
PM invasion on imaging *		
No	438 (83.7)	449 (85.4)
Suspicious	85 (16.3)	77 (14.6)
LN metastasis on imaging ^†^		
No	387 (74.0)	393 (74.7)
Suspicious	136 (26.0)	133 (25.3)
Pelvic lymphadenectomy		
No	1 (0.2) ^§^	1 (0.2) ^§^
Yes	522 (99.8)	525 (99.8)
Para-aortic lymphadenectomy		
No	405 (77.4)	414 (78.7)
Sampling/Dissection	118 (22.6)	112 (21.3)
Pathologic cervical mass size, mm ^‡^	28.0 (0–110.0)	26.0 (0–110.0)
No residual tumor	64 (12.2)	73 (13.9)
<20	114 (21.8)	120 (22.8)
≥20 and <40	206 (39.4)	200 (38.0)
≥40	139 (26.6)	133 (25.3)
Pathologic risk factors		
PM invasion	89 (17.0)	83 (15.8)
LN metastasis	137 (26.2)	123 (23.4)
Resection margin involvement	16 (3.1)	12 (2.3)
LVSI	227 (43.4)	209 (39.7)
Invasion depth ≥ 1/2	300 (57.4)	289 (54.9)
Adjuvant treatment		
No	229 (43.8)	247 (47.0)
Radiation only	86 (16.4)	83 (15.8)
CCRT	208 (39.8)	196 (37.3)

Presented with *n* (%) or median (range). Abbreviations: CA-125, cancer antigen 125; CEA, carcinoembryonic antigen; CCRT, concurrent chemoradiation therapy; CT, computed tomography; FIGO, International Federation of Gynecology and Obstetrics; LN, lymph node; LVSI, lymphovascular space invasion; MRI, magnetic resonance imaging; PET, positron emission tomography; PM, parametrial; SCC, squamous cell carcinoma antigen. * Measured by preoperative MRI; ^†^ Evaluated by MRI, CT, or PET/CT; ^‡^ Measured on the uterine specimen; ^§^ These two patients received conization and had no residual tumor without any suspicious LNs on preoperative imaging. Missing data: ^a^ 87; ^b^ 46; ^c^ 323; ^d^ 67; ^e^ 41; ^f^ 343.

**Table 2:** Summary of the selected variables using exhaustive variable selection methods.

Variables	PFS Set (*n* = 523)		OS Set (*n* = 526)	
	OR	90% CI	OR	90% CI
Surgical approach: Laparosocpy vs. Open	0.856	0.805–0.911		
CEA, ng/mL	0.994	0.991–0.997	0.997	0.995–0.999
SCC, ng/mL	0.995	0.992–0.997	0.995	0.993–0.997
Preoperative conization: Yes vs. No	1.091	1.022–1.166		
2009 FIGO stage, IB2 vs. IB1	0.908	0.832–0.991		
LN metastasis on imaging *: Suspicious vs. No	0.924	0.862–0.991	0.919	0.872–0.969
CA-125, IU/mL			0.999	0.998–1.000
Cervical mass size by MRI: ≥20 mm vs. <20 mm			0.942	0.899–0.986
Histologic type: Squamous vs. Non-squamous			1.073	1.021–1.127

Only variables with *p* < 0.1 are presented. Abbreviations: CA-125, cancer antigen 125; CEA, carcinoembryonic antigen; CI, confidence interval; CT, computed tomography; FIGO, International Federation of Gynecology and Obstetrics; LN, lymph node; MRI, magnetic resonance imaging; OR, odd ratio; PET, positron emission tomography; SCC, squamous cell carcinoma antigen. * Evaluated by MRI, CT, or PET/CT.

## Data Availability

The data presented in this study are also available on request from the corresponding authors.

## Supplementary Materials

The following are available online at https://www.mdpi.com/article/10.3390/cancers13153709/s1, Figure S1: Importance values of predictors from the machine learning algorithms: (**A**) 5-year PFS rate; (**B**) 5-year OS rate, Table S1: List of machine learning methods used in this study, Table S2: Clinicopathologic characteristics of study population.
