# Supplementary material for: Machine Learning Models to Predict Survival Outcomes According to the Surgical Approach of Primary Radical Hysterectomy in Patients with Early Cervical Cancer
Source: Cancers (Basel). 2021 Jul 23;13(15):3709. doi: 10.3390/cancers13153709 (PMC8345043; doi:10.3390/cancers13153709)
Supplement: Supplementary file 1 [file cancers-13-03709-s001.zip › cancers-1286711-supplementary.pdf]

Table 1. List of machine learning methods used in this study.

Method	Package	Version
Random forest	randomForest	4.6-14
Support Vector Machine	e1071	1.7-4
Decision Tree	C50	0.1.3.1
Neural Network	nnet	7.3-14
K-Nearest Neighbors	caret	6.0-86
Naïve Bayes	caret	6.0-86
Gradient Boosting	gbm	2.1.8
eXtreme Gradient Boosting	xgboost	1.3.2.1

Table 2. Clinicopathologic characteristics of study population.

**Characteristics**	**All patients (*n*=1056, %)**
Age, years	48.0 (24.6–87.0)
Surgical approach	
Open surgery	539 (51.0)
Laparoscopy	517 (49.0)
2009 FIGO stage	
IB1	878 (83.1)
IB2	178 (16.9)
Histologic type	
Squamous cell carcinoma	744 (70.5)
Adenocarcinoma	263 (24.9)
Adenosquamous carcinoma	49 (4.6)
Preoperative conization	
No	709 (67.1)
Yes	347 (32.9)
Preoperative tumor markers	
CEA, ng/ml [a]	1.3 (0.1–210.0)
SCC, ng/ml [b]	1.1 (0.1–118.7)
CA-125, IU/ml [c]	12.0 (0.9–506.8)
Cervical mass size by MRI, mm	22.0 (0–82.0)
No residual tumor	301 (28.5)
<20	164 (15.5)
≥20 and <40	392 (37.1)
≥40	199 (18.8)
PM invasion on imaging*	
No	798 (75.6)
Suspicious	258 (24.4)
LN metastasis on imaging†	
No	902 (85.4)
Suspicious	154 (14.6)
Pelvic lymphadenectomy	
No	11 (1.0)
Yes	1045 (99.0)
Para-aortic lymphadenectomy	
No	862 (81.6)
Sampling/Dissection	194 (18.4)
Pathologic cervical mass size, mm‡	27.0 (0–110.0)
No residual tumor	134 (12.7)
<20	248 (23.5)
≥20 and <40	402 (38.1)
≥40	272 (25.8)
Pathologic risk factors	

PM invasion	152 (14.4)
LN metastasis	221 (20.9)
Resection margin involvement	30 (2.8)
LVSI	405 (38.4)
Invasion depth ≥ 1/2	574 (54.4)
Adjuvant treatment	
No	497 (47.1)
Radiation only	193 (18.3)
CCRT	366 (34.7)

Presented with n (%) or median (range). Abbreviations: CA-125, cancer antigen 125; CEA, carcinoembryonic antigen; CCRT, concurrent chemoradiation therapy; CT, computed tomography; FIGO, International Federation of Gynecology and Obstetrics; LN, lymph node; LVSI, lymphovascular space invasion; MRI, magnetic resonance imaging; PET, positron emission tomography; PM, parametrial; SCC, squamous cell carcinoma antigen. *Measured by preoperative MRI; †Evaluated by MRI, CT, or PET/CT; ‡Measured on the uterine specimen. Missing data: [a]353; [b]109; [c]659.

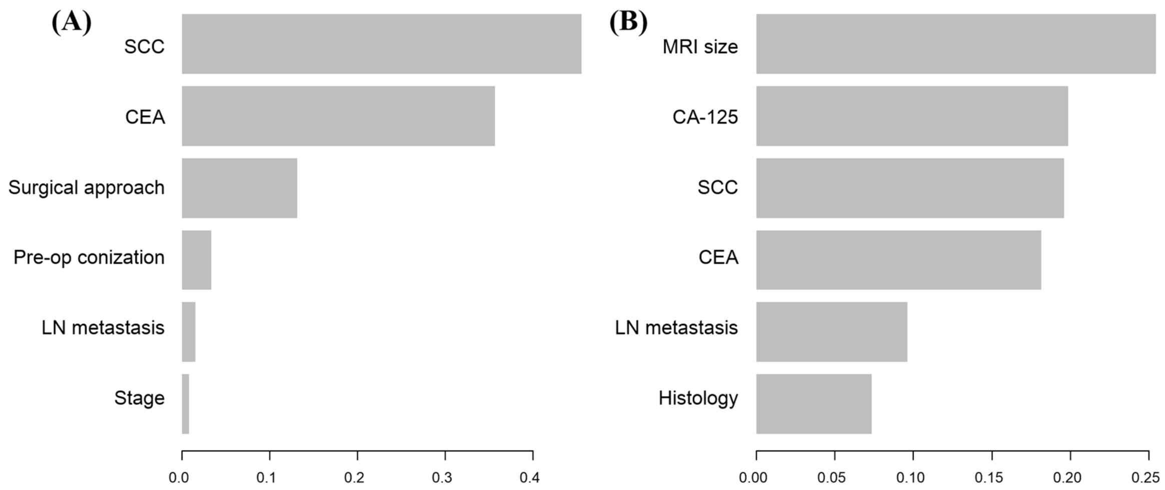

Figure S1: Importance values of predictors from the machine learning algorithms: (A) 5-year PFS rate; (B) 5-year OS rate
